# Viable CAR T-cells remain detectable in cerebrospinal fluid in patients with grade ≥3 ICANS despite corticosteroid therapy

**DOI:** 10.3389/fonc.2026.1842487

**Published:** 2026-06-11

**Authors:** Alexander Casimir Angleitner, Markus Maulhardt, Gerald Wulf, Judith Büntzel

**Affiliations:** Department of Hematology and Oncology, University Medical Center Göttingen, Göttingen, Germany

**Keywords:** CAR T-cells, CRS, CSF, ICANS, neurotoxicity

## Abstract

**Background:**

Immune effector cell-associated neurotoxicity syndrome (ICANS) is a severe complication of CAR T-cell therapy. Although CAR T-cells have been detected in cerebrospinal fluid (CSF), their viability remains unknown.

**Methods:**

We retrospectively analyzed all patients with grade ≥3 ICANS after commercial CAR T-cell therapy (February 2022 – September 2025) who underwent lumbar puncture (n=13). CSF was analyzed by flow cytometry including 7-AAD staining for CAR T-cell viability. Viable CAR T-cells were defined as CAR^+^CD3^+^7-AAD^−^ lymphocytes. A systematic literature review identified published cases with CSF CAR T-cell detection.

**Results:**

Among 133 CAR T-cell recipients, 28 developed grade ≥3 ICANS and 13 underwent lumbar puncture (total 22 samples). Viability testing was feasible in 11 samples; all contained viable CAR T-cells (median 75%, range 27–99%), which remained detectable up to day +98 despite corticosteroid therapy. Median CSF CAR T-cell count was 4.3/µL (IQR 1.7–18.1). Protein and albumin were elevated in most patients, indicating blood–CSF barrier disruption. A literature review identified 17 published cases reporting CSF findings after CAR T-cell therapy.

**Conclusion:**

To our knowledge, this is the first study demonstrating that CAR T-cells in the CSF are viable and remain detectable despite corticosteroid therapy. These findings demonstrate the presence of viable CAR T cells in the CSF of patients with ICANS and warrant prospective functional studies.

## Introduction

Chimeric antigen receptor (CAR) T-cell therapy has markedly improved outcomes across hematologic malignancies (1–5), but remains limited by potentially severe immune effector cell-associated neurotoxicity syndrome (ICANS). However, the biological mechanisms underlying ICANS remain incompletely understood, particularly the role of CAR T-cells within the central nervous system (CNS) (6, 7). Berger et al. recently demonstrated CAR T-cell kinetics in CSF by digital PCR (8), but systematic assessment of CAR T-cell viability has not been reported. This question is particularly relevant given that corticosteroids – the mainstay of ICANS treatment – might affect CAR T-cell survival and function.

To address this gap, we conducted a systematic viability-based analysis of CSF CAR T-cells in a consecutive single-center cohort of patients with grade ≥3 ICANS and contextualized our findings through a comprehensive literature review.

## Methods

### Study design and patients

This retrospective single-center study was conducted at the University Medical Center Göttingen in accordance with the Declaration of Helsinki and approved by the local ethics committee (approval number 26/3/25). We identified all consecutive patients who developed grade ≥3 ICANS according to ASTCT consensus criteria after commercial CAR T-cell therapy between February 2022 and September 2025 (n=28). Lumbar puncture (LP) was performed in a subset of patients based on clinical indication (n=13), primarily to further evaluate neurological symptoms and to exclude infectious or other alternative causes. Contraindications to LP included anticoagulation, thrombocytopenia, or other conditions associated with increased procedural risk. CRS and ICANS were graded according to the ASTCT consensus criteria (9).

### CSF analysis

CSF was obtained when clinically indicated during neurological toxicity. Routine analyses included cell count, protein, albumin, and lactate. When sufficient sample volume was available, flow cytometry was performed on a DxFlex flow cytometer (Beckman Coulter, Brea, CA, USA). Cell viability was assessed using 7-AAD staining with Stem-Kit reagents (Stem-Kit reagents, Immunotech SAS, Marseille, France). Viable CAR T-cells were defined as CAR^+^CD3^+^7-AAD^−^ lymphocytes using a sequential gating strategy, including exclusion of debris and doublets, identification of lymphocytes based on CD45 expression and side scatter, and viability assessment via 7-AAD. CD4^+^ and CD8^+^ CAR T-cell subsets were further characterized. Detailed protocols and gating strategies are provided in the **Supplementary Material**.

### Systematic literature review

We conducted a PubMed search on September 2, 2025 using the string: ((car t cell) AND ((cerebrospinal fluid) OR (CSF))). Filters were applied for case reports and case series. The search yielded 38 records; 4 were identified as relevant after screening titles and abstracts. Manual reference screening identified additional cases.

### Statistical analysis

Descriptive statistics were used. Continuous variables are summarized as median (IQR or range). Analyses were conducted using Python 3.11.

## Results

### Patient characteristics

Between February 2022 and September 2025, 133 patients received CAR T-cell therapy at our center. Of these, 28 consecutive patients (21%) developed grade ≥3 ICANS and 13 patients underwent lumbar puncture. Median age was 57 years (range 30–72); 8 were male. Diagnoses included DLBCL (n=9), MCL (n=2), B-ALL (n=1), PMBL (n=1). Patients received axicabtagene ciloleucel (axi-cel; n=8), brexucabtagene autoleucel (brexu-cel; n=2), tisagenlecleucel (tisa-cel; n=2), and lisocabtagene maraleucel (liso-cel; n=1). All patients received lymphodepleting chemotherapy with fludarabine and cyclophosphamide. Additional demographic and disease-specific characteristics are summarized in **Supplementary Table 1**.

### Toxicities

All 13 patients developed grade ≥3 ICANS and mostly mild to moderate CRS (median grade 2, IQR 2–3). ICU admission occurred at a median of 5 days (IQR 4–6) post-infusion, with median ICU stay of 10 days (IQR 3–12). The median time between ICU admission and lumbar puncture was 2 days (IQR 0–4 days). All patients received high-dose corticosteroids.

### CSF findings

**Figure 1:**
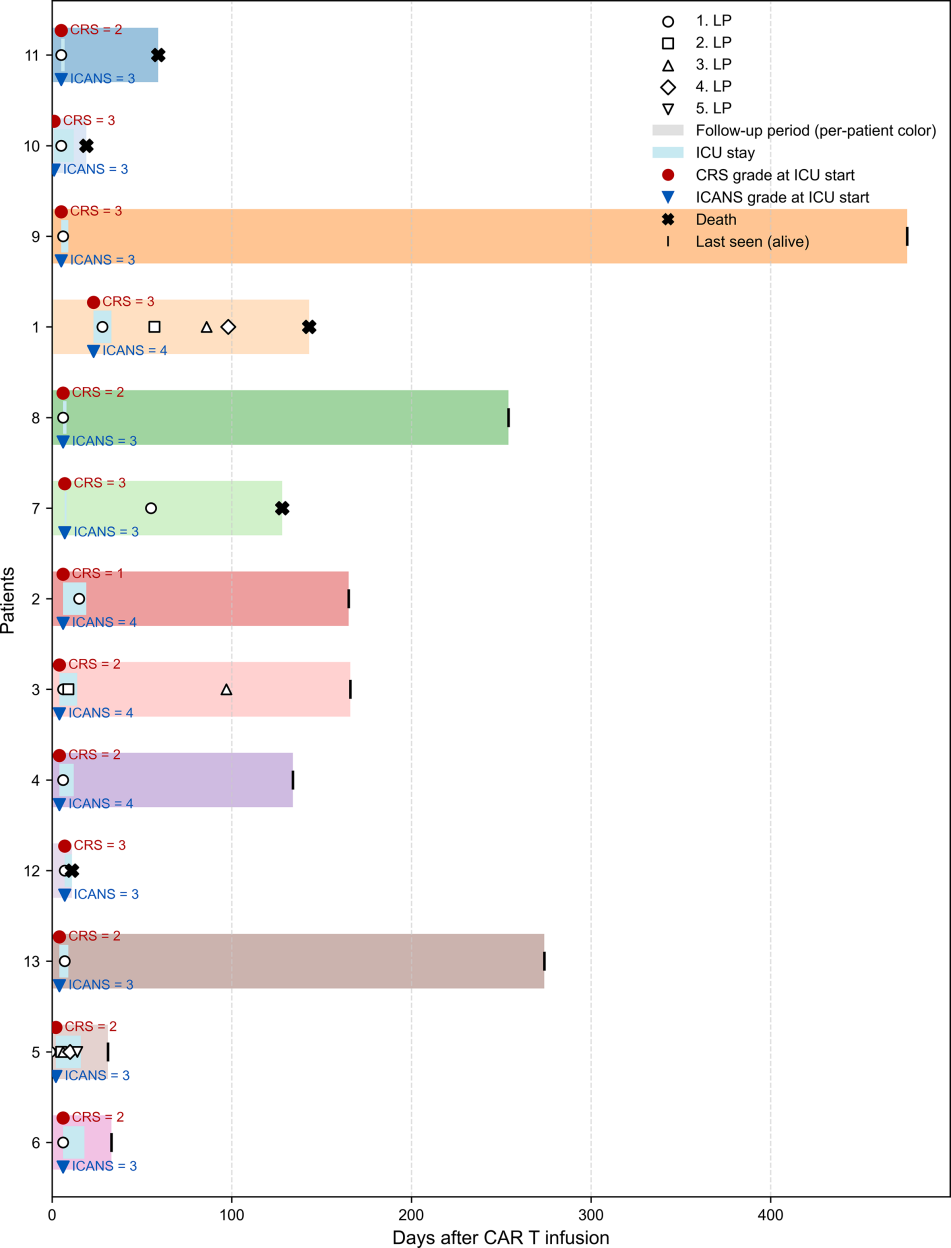
Longitudinal clinical course and CSF detection of CAR T-cells in patients with grade ≥3 ICANS. Swimmer plot illustrating the longitudinal clinical course of patients with grade ≥3 immune effector cell-associated neurotoxicity syndrome (ICANS) undergoing lumbar puncture after CAR T-cell therapy. Horizontal bars represent the follow-up period after CAR T-cell infusion. Symbols indicate intensive care unit (ICU) stay, timing of lumbar punctures, cytokine release syndrome (CRS), ICANS severity, and detection of CAR T-cells in CSF. Black crosses denote death and vertical black lines indicate last follow-up. Viable CAR T-cells remained detectable in CSF for up to 98 days after CAR T-cell infusion despite corticosteroid therapy.

A total of 22 CSF samples were obtained from 13 patients (median 1 sample per patient, range 1–4). CAR T-cells were detected in 12 samples from 8 patients (**Figure 1**). Viability testing was feasible in 11 of 12 samples; in one sample, viability assessment was not performed, and the exact reason could not be retrospectively determined. This sample did not differ from the remaining evaluable samples in terms of cell count or timing of CSF collection. All evaluable samples contained viable CAR T-cells, with a median viability of 75% (range 27–99%). CAR T-cells remained detectable in CSF + 98 days post-infusion despite ongoing high-dose corticosteroid therapy.

Median CSF CAR T-cell count was 4.3/µL (IQR 1.7–18.1). Routine CSF parameters frequently indicated blood–CSF barrier dysfunction: median protein 997 mg/L (IQR 685–2752), albumin 852 mg/L (IQR 600–3113), and lactate 2.3 mmol/L (IQR 1.6–5.1).

### Outcomes

Five patients died during follow-up (median follow-up 134 days, IQR 46–166). Causes of death included HHV-6 encephalitis (n=1), relapse (n=3), and unknown causes (n=1). The patient with HHV-6 encephalitis had detectable CSF CAR T-cells at the time of diagnosis.

### Published cases

Our literature review identified 17 published cases reporting CSF analyses after CAR T-cell therapy, including several cases with detectable CSF CAR T-cells (**Supplementary Tables 2**, **3**. Diagnoses included primary CNS lymphoma (n=6), secondary CNS lymphoma (n=4), and systemic lymphomas with CNS involvement (n=7). ICANS occurred in 13 of the 17 patients (76%; grade 1-4), while 4 patients had CSF CAR T-cells in the absence of neurotoxicity (e.g., CNS myeloma without ICANS). None of the studies reported systematic viability assessment.

Due to substantial heterogeneity in sampling timing, clinical indications for lumbar puncture (ICANS, relapse or monitoring), and detection methods across reports, we did not perform quantitative comparisons between our cohort and published cases. A descriptive summary of individual patient data is provided in **Supplementary Tables 2** and **3**.

## Discussion

This study provides the first systematic evidence that CAR T-cells in the CSF are viable and remain detectable for weeks to months after infusion, even under high-dose corticosteroid therapy. Our findings extend previous observations of CAR T-cell presence in CSF, as summarized in the supplementary case analyses (**Supplementary Table 2**), by demonstrating that these cells remain viable, although viability alone does not establish functional activity or a causal contribution to ICANS pathophysiology.

The continued detectability of viable CAR T-cells despite corticosteroids is consistent with reports indicating limited effects of steroids on CAR T-cell expansion (10, 11). The frequent evidence of blood–CSF barrier disruption supports a model in which systemic inflammation and localized cytokine activity facilitate CAR T-cell trafficking into the CNS (12). While clinical and translational data predominantly support cytokine-mediated mechanisms as the main drivers of ICANS, including endothelial activation and blood–brain barrier disruption (6, 7), direct on-target effects remain a theoretical possibility. In this context, CD19 expression on brain mural cells has been proposed as a potential off-tumor target, although this has not been demonstrated in clinical settings (13). Notably, patients with preexisting frontal lobe impairment may develop ICANS earlier, potentially reflecting increased vulnerability to cytokine-mediated neurotoxicity (14).

The fatal HHV-6 encephalitis in one patient underscores an important clinical consideration: infectious etiologies may mimic or coexist with ICANS (15). The presence of viable CAR T-cells in CSF should not preclude thorough infectious workup, particularly in patients receiving prolonged corticosteroids.

Our study has limitations. The retrospective design and small sample size reflect the rarity of grade ≥3 ICANS with available CSF. Lumbar punctures were clinically indicated, introducing selection bias toward patients with more severe neurological manifestations and those in whom the procedure was feasible. As a result, findings may not be fully generalizable to the broader ICANS population. CSF sampling was largely cross-sectional, precluding assessment of temporal dynamics. Although 7-AAD negativity indicates membrane integrity, we did not perform functional assays; viability does not equate to cytotoxic activity. Finally, heterogeneity of published cases limited quantitative comparisons.

Despite these limitations, this study represents the largest systematic viability-based assessment of CSF CAR T-cells to date. The consistent detection of viable CAR T-cells in CSF samples despite corticosteroid exposure provides additional descriptive evidence that CAR T-cells may remain detectable within the CNS during ICANS.

## Conclusion

CAR T-cells remain viable in the CSF during grade ≥3 ICANS despite high-dose corticosteroid therapy. Whether these cells retain functional activity requires further investigation. Standardized prospective studies integrating functional analyses and viral diagnostics are needed to define the role of CNS-resident CAR T-cells in neurotoxicity.

## Data Availability

The raw data supporting the conclusions of this article will be made available by the authors, without undue reservation.
