# Supplementary material for: Viable CAR T-cells remain detectable in cerebrospinal fluid in patients with grade ≥3 ICANS despite corticosteroid therapy
Source: Front Oncol. 2026 Jun 11;16:1842487. doi: 10.3389/fonc.2026.1842487 (PMC13293836; doi:10.3389/fonc.2026.1842487)
Supplement: Supplementary file 1 [file Table1.docx]

Supplementary Table 1. Baseline characteristics of the study cohort

| Variable | Value |
| --- | --- |
| Number of patients | 13 |
| Age, years; median (range) | 57 (30-72) |
| Sex, female/male | 5 (38%) / 8 (62%) |
| Diagnosis | |
| DLBCL | 9 (69%) |
| MCL | 2 (15%) |
| B-ALL | 1 (8%) |
| PMBL | 1 (8%) |
| Disease Stage (DLBCL) | |
| IIA | 1 |
| IIE | 1 |
| IIIA | 2 |
| IIIE | 1 |
| IV | 1 |
| IVB | 3 |
| CAR T-cell product | |
| axi-cel | 8 (62%) |
| brexu-cel | 2 (15%) |
| tisa-cel | 2 (15%) |
| liso-cel | 1 (8%) |
