# Supplementary material for: Viable CAR T-cells remain detectable in cerebrospinal fluid in patients with grade ≥3 ICANS despite corticosteroid therapy
Source: Front Oncol. 2026 Jun 11;16:1842487. doi: 10.3389/fonc.2026.1842487 (PMC13293836; doi:10.3389/fonc.2026.1842487)
Supplement: Supplementary file 2 [file Table2.docx]

# *Supplementary Table 2. Clinical Information of Reported Cases*

| Author (Year) | Journal | Country/Center | Patient ID (Lit.) | Age | Sex | Diagnosis | Disease details / Prior therapy | CAR-T Product |
| --- | --- | --- | --- | --- | --- | --- | --- | --- |
| De Philippis et al. (2025) | Cytotherapy | Italy – San Raffaele Hospital, Milan | Lit-01 | 44 | Male | Mantle Cell Lymphoma (TP53+) | MIPI 5.2 (low); refractory to chemo + ibrutinib; bridging with ibrutinib (no control achieved) | brexu-cel (Tecartus) |
| Perriello et al. (2023) | Am J Hematol | Italy – Perugia University Hospital | Lit-02 | 63 | Male | Primary cardiac DLBCL (ABC subtype, c-MYC+) | R-COMP (PR, early relapse); salvage R-DHAOX (no response); bridging Pola-Benda-R (CR before CAR-T) | tisa-cel (Kymriah) |
| Hu et al. (2016) | J Hematol Oncol | China – Zhejiang University, Hangzhou | Lit-03 | 43 | Female | BCR/ABL p210+ r/r ALL with CNS involvement | VDCLP, Hyper-CVAD A/B, IAE, MTX + L-ASP, IA, EA + DXM; ~15× intrathecal; TKI; lymphodepletion FC | Autologous anti-CD19 CAR-T (FMC63 scFv; 4-1BB + CD3ζ) |
| Li et al. (2020) | Front Oncol | China – Tongji Hospital, Wuhan | Lit-04 | 39 | Male | Secondary CNS DLBCL, non-GCB | R-CHOP ×6 (CR), CNS relapse, HD-MTX + rituximab + temozolomide (PD) | Autologous anti-CD19 + anti-CD22 CAR-T cocktail |
| Li et al. (2020) | Front Oncol | China – Tongji Hospital, Wuhan | Lit-05 | 60 | Male | Secondary CNS DLBCL, GCB | RT ×13F, R-CHOP ×8 (CR), lenalidomide; CNS relapse; R-ICE ×3 (PD), R-MA ×3 (PD) | CAR19/22 cocktail |
| Li et al. (2020) | Front Oncol | China – Tongji Hospital, Wuhan | Lit-06 | 38 | Male | Secondary CNS DLBCL, GCB | R-DA-EPOCH, R-HD-MTX, R-DHAP, R-HD-AraC ×2 (all PD); systemic + CNS progression | CAR19/22 cocktail |
| Li et al. (2020) | Front Oncol | China – Tongji Hospital, Wuhan | Lit-07 | 18 | Female | Secondary CNS DLBCL, NOS | R-CHOP ×5 (CR), R-DA-EPOCH, R-HD-MTX, RT ×5F; CNS relapse | CAR19/22 cocktail |
| Li et al. (2020) | Front Oncol | China – Tongji Hospital, Wuhan | Lit-08 | 49 | Female | Primary CNS DLBCL, GCB | HD-MTX ×3 (PR), R-HD-MTX ×2 (PR→PD), progression | CAR19/22 cocktail |
| Siddiqi et al. (2021) | Blood Advances | USA / City of Hope, CA | Lit-09 | 53 | Female | Primary CNS lymphoma (DLBCL) | High-dose methotrexate-based chemotherapy | Autologous CD19-CAR T (CD28 costim., EGFR marker) |
| Siddiqi et al. (2021) | Blood Advances | USA / City of Hope, CA | Lit-10 | 53 | Female | Primary CNS lymphoma (DLBCL) | Pomalidomide | Autologous CD19-CAR T (CD28 costim., EGFR marker) |
| Siddiqi et al. (2021) | Blood Advances | USA / City of Hope, CA | Lit-11 | 47 | Female | Primary CNS lymphoma (DLBCL) | 12 prior lines, brain radiation, steroids | Autologous CD19-CAR T (CD28 costim., EGFR marker); CSF+: CAR-T im Liquor |
| Siddiqi et al. (2021) | Blood Advances | USA / City of Hope, CA | Lit-12 | 49 | Female | Primary CNS lymphoma (DLBCL) | Multiple prior therapies; no bridging | Autologous CD19-CAR T (CD28 costim., EGFR marker) |
| Siddiqi et al. (2021) | Blood Advances | USA / City of Hope, CA | Lit-13 | 42 | Female | Primary CNS lymphoma (DLBCL) | Brain radiation, steroids | Autologous CD19-CAR T (CD28 costim., EGFR marker) |
| Zhang et al. (2021) | Frontiers in Immunology | China – First Affiliated Hospital of Soochow University, Suzhou | Lit-14 | 56 | Male | Isolated CNS Multiple Myeloma (κ light chain) | Diagnosed 2014; ≥5 prior therapy lines (proteasome inhibitors, IMiDs, daratumumab, cytarabine, methotrexate, cisplatin, etoposide, cyclophosphamide); refractory CNS/spinal involvement since 2017 | Autologous BCMA-directed CAR-T cells (clinical trial NCT03196414) |
| Pennese et al. (2023) | Frontiers in Immunology | Italy – Hematology Unit, Spirito Santo Hospital, Pescara / University of Chieti-Pescara | Lit-15 | 55 | Female | Diffuse large B-cell lymphoma (DLBCL), refractory | Multiple prior lines: R-COMP (rituximab, cyclophosphamide, vincristine, liposomal doxorubicin, prednisone), R-DHAP (rituximab, dexamethasone, cytarabine, cisplatin), R-IEV (rituximab, ifosfamide, epirubicin, etoposide) – refractory disease, not eligible for auto-HCT | axi-cel (Yescarta) |
| Katsin et al. (2024) | Frontiers in Immunology | Belarus – Vitebsk Regional Clinical Cancer Centre; Belarusian Research Center for Pediatric Oncology, Hematology and Immunology, Minsk; Institute of Bioorganic Chemistry, National Academy of Sciences of Belarus | Lit-16 | 39 | Male | Relapsed/refractory stage IV Primary Mediastinal B-Cell Lymphoma (PMBCL) | Refractory to R-DA-EPOCH, R-MACOP-B, and Nivolumab + Brentuximab vedotin; bulky mediastinal disease (170 mm), pleural, lung, BM and LN involvement; high IPI; CAR-HEMATOTOX score 2 | Autologous second-generation CD19 CAR-T cells (FMC63 scFv, IgG4 hinge, CD28 TM, 4-1BB, CD3ζ; EGFRt marker; expanded with IL-7/IL-15) |
| Maulhardt et al. (2025) | Hemasphere | Multicenter (Germany/Switzerland) | Lit-17 | NR | NR | Relapsed/Refractory Multiple Myeloma with CNS involvement | heavily pretreated r/r MM with prior CNS manifestation | ide-cel (Abecma) |
