# Supplementary material for: Viable CAR T-cells remain detectable in cerebrospinal fluid in patients with grade ≥3 ICANS despite corticosteroid therapy
Source: Front Oncol. 2026 Jun 11;16:1842487. doi: 10.3389/fonc.2026.1842487 (PMC13293836; doi:10.3389/fonc.2026.1842487)
Supplement: Supplementary file 3 [file Table3.docx]

Supplementary Table 3. CSF/LP Findings in Reported Cases

| Patient ID | Day of LP | CAR-T detection (Y/N, %, abs.) | Viability reported (Y/N) | CSF findings (Protein, cytokines, pleocytosis) | ICANS grade / symptoms | CRS grade | Outcome |
| --- | --- | --- | --- | --- | --- | --- | --- |
| Lit-01 | +10 | PB only – no CSF analysis | N | No LP performed | ICANS 3 (confusion, aphasia) | CRS 2 (d+4) | Alive, 1y CR |
| Lit-01 | +30 | Y (3.8/µL) | N | NR | Persistent fever, fatigue | Late CRS | – |
| Lit-01 | +58 | Y (73/µL, ~9%) | N | Protein↑, Glucose↓, OP 18mmHg, infection– | Headache, dysarthria, gait instability | – | – |
| Lit-01 | +68 | N (0) | N | NR | No new ICANS | – | – |
| Lit-02 | +10 | Y (~4% T cells CAR⁺, abs 797/µL) | N | Protein 1450 mg/L↑, pleocytosis, cytokines↑ | ICANS 3 (confusion, aphasia, palsy) | CRS 1 | Alive d+17, later relapse |
| Lit-02 | +17 | Y (~50% T cells CAR⁺, abs 267/µL) | N | Protein 198 mg/L, cytokines↑ | Improved under steroids | – | Temporary improvement |
| Lit-02 | +79 | NR, CD79b+ relapse | – | Malignant cells detected (CD19– escape) | Relapse, not ICANS | – | Died 4mo post CAR-T (PD) |
| Lit-03 | +5 | Y (CAR+ detected, no abs) | N | Cytokines↑, pleocytosis, protein↑ | Headache, seizure, disorientation | CRS 2 | CR, CNS cleared, alive |
| Lit-04 | ~+1–2 mo | Y after PD (ddPCR copies) | N | CSF+ lymphoma cells at PD | None | CRS 1 | PR → PD 3mo; stable w/ RT+PD1, alive 16mo |
| Lit-05 | + ~4 mo negative +~8–9 mo positive (relapse) | Y (88.8% lymphocytes; 40k copies) | N | CSF MRD– → relapse; cytokines↑ | ICANS 1 (numbness) | CRS 1 | PR→CR 6mo→relapse 8mo; stable 16mo |
| Lit-06 | NR | Y (peak levels, % NR) | N | Cytokines↑, IL-6/ferritin highest | ICANS 4 (somnolence, delirium, dysmnesia, reversible) | CRS 2 | CR; ASCT+CAR-T; remission 14mo |
| Lit-07 | NR | Not clearly reported | N | NR | None | CRS 1 | PR→PD 3mo; RT; alive 7mo |
| Lit-08 | NR | Y (CAR19=55, CAR22=93 copies) | N | NR | None | CRS 1 | PR; ASCT+CAR-T; CR 6mo |
|  |  |  |  |  |  |  |  |
| Lit-09 | No LP performed | NA | NA | NA | Grade 3 NT – headache (3), agitation (1), restlessness (1) | 2 | CR; progressed at day 273, alive |
| Lit-10 | No LP performed | NA | NA | NA | Grade 2 NT – headache (2), dizziness (2), memory impairment (1) | 1 | SD; off protocol, alive on pomalidomide |
| Lit-11 | Day 0, 7, 14 | Yes – CAR-T detected in CSF by flow cytometry (EGFR+); %/abs. not quantified in text | No | No further details (only CAR-T cells shown in Fig. 1D) | Grade 1 NT – tremor (1), dysarthria (1), hallucinations (1) | 1 | SD; later progressed, lost to follow-up |
| Lit-12 | No LP performed | NA | NA | NA | Grade 1–2 NT – concentration impairment (1), dysphasia (1) | 2 | CR; alive, ongoing remission (day 520) |
| Lit-13 | No LP performed | NA | NA | NA | Grade 1 NT – seizure (1), dizziness (1) | 1 | CR; alive, off protocol, on lenalidomide |
| Lit-14 | +9 | Yes — clear CAR transgene in CSF (peak; explicit in text and shown in Fig. 2) | NR | IL-6 ~5,000 pg/mL (from 10 pg/mL baseline), ↑IFNγ, IL-2, IL-10; pleocytosis; ↑LDH; ↑protein | Grade 4: stupor, aphasia, pupillary asymmetry, loss of light reflex, obtundation, stiff neck | Not reported; fever + hypotension ~6 h post-infusion (tocilizumab not given) | IV methylprednisolone + mannitol started. |
| Lit-14 | +12 | Yes — CAR-T signal in CSF, lower than D+9 | NR | IL-6 still markedly elevated (per curve) | Improving from nadir | — | Ongoing monitoring. |
| Lit-14 | +15 | NR | NR | IL-6 still high → first intrathecal dexamethasone (5 mg); subsequent IL-6 drop; WBC shift from neutrophil- to monocyte-predominant | Clinical improvement | — | Stabilization. |
| Lit-14 | +18 | NR | NR | Further IL-6 decline after second IT-DEX; CSF leukocytes persist but decreasing | Further improvement | — | Stabilization. |
| Lit-14 | +23 | NR | NR | No monoclonal plasma cells; normal κ/λ ratio | By Day 24: normal mental status | — | Partial response; >50% MRI lesion reduction by Day 30; response sustained 5.5 months. |
| Lit-15 | ~+6 | Yes – 4 cells/µL, 97.4% CAR-T+ | NR | Only T cells present; no infection, no lymphoma involvement | ICANS IV (coma, ICU, high-dose steroids + Anakinra) | Already CRS grade 3 (fever, cardiopulmonary instability on Day +1) | Severe ICANS; ICU treatment started |
| Lit-15 | ~+13 | Yes – CAR-T still positive (quant. not given, text: remained positive) | NR | Steroids given intrathecally; no malignant cells/infection | ICANS improving | – | Stabilization |
| Lit-15 | ~Day +20 | Yes – CAR-T still positive | NR | No malignant cells, CSF otherwise unremarkable | Further improvement, ICANS regressing | – | Clinical recovery continuing |
| Lit-15 | ~+30 | No – CAR-T negative in CSF | NR | No infection, no lymphoma; CSF clear | ICANS resolved (ended Day +24) | – | PET/CT PR at Day +30; long-term CR at 15 months |
| Lit-16 | +14 | Yes – CAR-T cells comprised 31.6% of leukocytes in CSF; CD4+/CD8+ ratio 54 | NR | Normal opening pressure; 2 WBC/µL total; no bacterial/viral infection; CSF otherwise unremarkable | ICANS Grade IV: coma, non-responsive to stimuli; occurred after initial Grade III deterioration despite IV dexamethasone | None reported (no concomitant CRS; patient never developed CRS) | Rapid improvement after IT MTX (15 mg), Ara-C (40 mg), dexamethasone (4 mg): Grade IV → II within 6 h, Grade 0 within 32 h; systemic steroids discontinued; sustained CR on PET-CT at 3 months, ongoing at 9 months |
| Lit-17 | +30 | Yes – BCMA CAR-T detectable in CSF | **NR** | 2.8 Zellen/µL, 17.9% CAR+ | None | NR | CNS response: PR→CR; serologic CR |
